# Multiple Gestations and Assisted Reproductive Technologies: Qualitative Study of the Discourse of Health Professionals in Spain

**DOI:** 10.3390/ijerph18116031

**Published:** 2021-06-03

**Authors:** Estefanía Jurado-García, Alicia Botello-Hermosa, Francisco Javier Fernández-Carrasco, Juan Gómez-Salgado, Nazaret Navas-Rojano, Rosa Casado-Mejía

**Affiliations:** 1 Department of Nursing, Escuela Universitaria de Osuna, University of Seville, 41640 Sevilla, Spain; 2 Department of Nursing, Faculty of Nursing, Physiotherapy and Podiatry, University of Seville, 41009 Sevilla, Spain; abotello@us.es (A.B.-H.); rcasado@us.es (R.C.-M.); 3 Department of Gynaecology and Obstetrics, Punta de Europa Hospital, 11207 Cádiz, Spain; franfernanca@hotmail.com; 4 Department of Nursing and Physiotherapy, Faculty of Nursing, University of Cadiz, 11009 Cádiz, Spain; 5 Department of Sociology, Social Work and Public Health, Faculty of Labour Sciences, University of Huelva, 21007 Huelva, Spain; jgsalgad@gmail.com; 6 Safety and Health Postgraduate Programme, Espíritu Santo University, Guayaquil 092301, Ecuador; 7 EIS Methods, Empresa de Base Tecnológica (Spin Off), University of Huelva, 21007 Huelva, Spain; nazaretnaro@gmail.com

**Keywords:** multiple pregnancy, assisted reproductive technologies (ART), embryo transfer, advanced maternal age, health system, maternal psychosocial consequences, parenting, perinatal outcomes, maternal and perinatal morbidity

## Abstract

Multiple gestations have become an increasing phenomenon that has impacted public health globally, largely due to the application of assisted reproductive technologies. The objective of this work was to find out the discourse that the health professionals involved in its follow-up have in our context. For this, a qualitative methodology was chosen, with semi-structured interviews recorded in audio, prior authorisation, and transcribed verbatim. It was based on a script designed for this purpose, with the following analysis categories: the current trend of multiple gestations, impact, and follow-up. The content analysis was based on the experiences, knowledge, and perceptions of the professionals interviewed. Professionals stated that the current socioeconomic and legal context hinders a single embryo transfer policy that decreases multiple gestation rates. They emphasised the importance of the psychic impact of such gestations on the couple, on the mother in particular, as well as the economic effect on families, health, and society in general. They expressed the need to create specific protocols to assist these gestations. Midwives, in particular, demanded that the health administration recognise and support the differentiated care they perform with this type of gestation. Work on specific models is needed to adequately size the impact of multiple gestations, as well as to generate social health policies that lead to co-responsible reconciliation measures that favour women having one pregnancy at a time.

## 1. Introduction

In the last fifty years, the incidence of multiple gestations has increased, acquiring epidemic dimensions, mainly due to delayed procreation and assisted reproductive technologies (ART). They have gone from representing 2% of births to 30–35% after the use of ART [1,2].

In this regard, it should be noted that much effort has been made to identify a correct algorithm that considers women’s age and ovarian reserve markers as a tool to optimise the initial dose of recombinant follicle stimulating hormone (rFSH) in intrauterine insemination cycles. However, according to the current available evidence, applying this algorithm with respect to women with polycystic ovary syndrome, especially those with elevated anti-Mullerian hormone, does not seem appropriate [3]. Likewise, the current trend is to perform assisted reproduction treatments associated with ovarian stimulation, but in the case of obese women, these require significantly higher amounts of gonadotropins to achieve the success rates of in vitro fertilisation, similar to those of women with normal weight [4].

Multiple gestations deserve special attention for their disproportionate contribution to maternal and perinatal morbidity, which has a special impact on public health [5]. They imply increased risks of maternal adverse outcomes such as hypertensive pregnancy states, gestational diabetes, bleeding, and postpartum depression (40% more likely), among others. They also involve foetal and neonatal risks such as higher rates of prematurity (50% of births occur before 37 weeks) or perinatal mortality and longer-term neurological developmental disorders (the risk of cerebral palsy is four times higher than in a single gestation) [6,7]. In addition, there are complications typical of twin pregnancy, i.e., growth discordance, intra-uterus foetal death of one of the twins, and twin-to-twin transfusion syndrome [8].

In gestations based on ART, the risk of adverse maternal and neonatal outcomes is further accentuated not only by the higher incidence of multiple pregnancy, the most common undesirable side effect, but also by the manipulation involved in these processes [9]. This has led to the introduction of elective policies for the transfer of individual embryos and declarations of consensus at the international level [10].

The impact of multiple gestations is not only on maternal (physical and emotional) [11] and neonatal health, but also on the health system itself (greater clinical and economic burden), families, and society in general [12]. In Europe, this impact differs among countries, and the number of foetuses in multiple gestation is one of the indicators of the European Perinatal Health Report (Euro-Peristat) programme to measure perinatal health in Europe [2].

This study was raised with the aim of understanding the experience and perception that the involved specialists have through their discourse, hoping to contribute to improve the integral attention to multiple gestations.

## 2. Materials and Methods

### 2.1. Study Design

This was a qualitative study within the hermeneutic phenomenological perspective, with the aim of knowing and understanding the discourses of Andalusian health professionals involved in the monitoring of multiple gestations.

### 2.2. Participants

The sampling was intentional, starting from two key informant professionals who facilitated the selection of other participants, resulting in a snowball sampling technique.

In total, eight professionals directly involved in the monitoring of multiple gestations in the city of Seville participated: 4 gynaecologists working in Assisted Reproductive Units (responsible for 30% of multiple gestations), and 4 primary care (PC) midwives, for their linkage to women throughout the pregnancy and postpartum process. The eight planned interviews were carried out until the saturation level was reached.

The targeting criteria were sex and professional category, which originated four profiles, resulting in two interviews per profile. In addition, service time, age, and scope of work were included as attributes that would make it easier to obtain greater discursive power (Table 1).

### 2.3. Data Collection

The fieldwork was carried out in the capital of the province of Seville, between January and February 2018. Semi-structured interviews were conducted.

An ad hoc script (Supplementary Materials S1 and S2) was used from the main study dimensions:

- Current trend of multiple gestations (embryo transfer policies).
- Impact and consequences on the family, society, and the health system.
- Monitoring of multiple gestations.
- Demands on health administration (emerging category).

It was the same script in the two groups, but some different questions were introduced to suit the idiosyncrasy of the assistance given by each group. The affected dimensions were *Current Trend of Multiple Gestations* with the question: “How do you address the issue of selective reduction?” in the script of gynaecologists and *Follow-up* with the question: “What strengths and weaknesses do you think the follow-up that is performed for pregnant women from primary care presents?” in that of midwives.

Two scripts designed for the purpose (see Supplementary Materials S1 and S2) were used, adapted to each professional category and based on the main dimensions of the study: current trend of multiple gestations (its relationship with ART and embryo transfer policies), monitoring of multiple gestations, and their impact, while also incorporating another emerging category of relevance and interest (demands to the health administration).

The interviews were recorded, lasting between 50 and 70 min. The exact date and place were set by the participants, with special consideration of the place, being free of interruptions, ensuring privacy and allowing them to be relaxed. Before starting each interview, the interviewer described the purpose of the study, explained the basic rules, and offered confidentiality guarantees.

To minimise the variability of the interview process, all of them were performed by the same researcher who took field notes from each of them with the aim of detecting and recording nonverbal language. The researcher applied summary and paraphrase techniques to cross-validate the collected information from the interviewees and minimise the possibility of data distortion. The transcripts were carried out by an expert in the field from outside the research team.

### 2.4. Data Analysis

Content analysis was used as an analysis technique to look closer at the experiences and knowledge of professionals, and to try to grasp the subjective meaning of their discourse regarding the categories of analysis raised [13]. This was carried out in terms of the main categories raised (current trend of multiple gestations, monitoring, and their impact) and incorporating another emerging category of relevance and interest (demands to the health administration). As an indicator of the validity of the results, a triple triangulation was performed that involved performing data triangulation, methodological (intra-method) triangulation, and triangulation of the research team.

Atlas.ti textual analysis software (Windows v8.0, Microsoft, Redmond, WA, USA) was used as an analysis tool. All transcripts of interviews were encoded, grouped by nodes or topics (categories), and text fragments were assigned to previously established categories.

Analysis of the interviews showed a series of arguments linked to the different categories of analyses created:

- Opinion on multiple gestations.
- Attribution of multiple gestations.
- Approach to embryo transfer policies.
- Circumstances, motivations, or needs to undergo an ART. Impact of ART on the couple, at the work and social level.
- Motivations for the delay of maternity
- Impact of multiple gestation.
- Follow-up of pregnant women from primary care.
- Emotional monitoring for women and couples undergoing ART.
- Negative situation in emotional follow-up.
- Follow-up in the postpartum.
- Maternal mental health during follow-up.
- Treatment of health administration of multiple gestations.
- Role of the professionals of the administration in the treatment.
- Suggestions.

This qualitative analysis has been carried out through a rooting criterion, hierarchically categorising the generated codes. In this way, the codes with the highest number of appointments have been prioritised. For its part, the marked cut-off point is located at the lower limit of the second quartile of the distribution, using the codes found in the first two quartiles of the appointment distribution for the analysis.

### 2.5. Ethical Considerations

This study adhered to the principles articulated in the Declaration of Helsinki, updated in 2013 in Brazil. To ensure anonymity, personal identifiable data were replaced by numbers. All the participants signed an informed consent. Authorisation was obtained from the Research Ethics Committee of the University Hospital Virgen Macarena in Seville, Spain, with code #09876, on 18 July 2014.

The data collected in the study will be treated with absolute confidentiality in accordance with the provisions of Spanish laws, specifically Organic Law 3/2018, of December 5th, on the Protection of Personal Data and Guarantee of Digital Rights, and Law 41/2002, of November 14th, basic regulation of the autonomy of the patient and rights and obligations in matters of information and clinical documentation.

## 3. Results

Data organisation made according to the categories of analysis facilitated the exposure of the results presented through the most relevant verbatims. Similarly, each category of analysis shows, through figures, the resulting cognitive maps from having raised issues to the participants to analyse these categories, complementing the information provided in the results.

### 3.1. Current Trend of Multiple Gestations (Embryo Transfer Policies)

There is a coincidence in the increase in multiple gestations, mostly due to the use of ART. Although the current trend is to try and decrease them, it does not seem so easily applicable due to hindering social and legal connotations.

It is considered that this may be due to variables such as the socioeconomic context of couples where multiple gestation is an advantage over only one (infertility treatments are very expensive and allowing more than one is highly unlikely), the employment situation of women (there is no real reconciliation that will protect them and shield their reproductive desires), and due to legal protection so as not to implement single transfer policies (Table 2).

**Figure 1:**
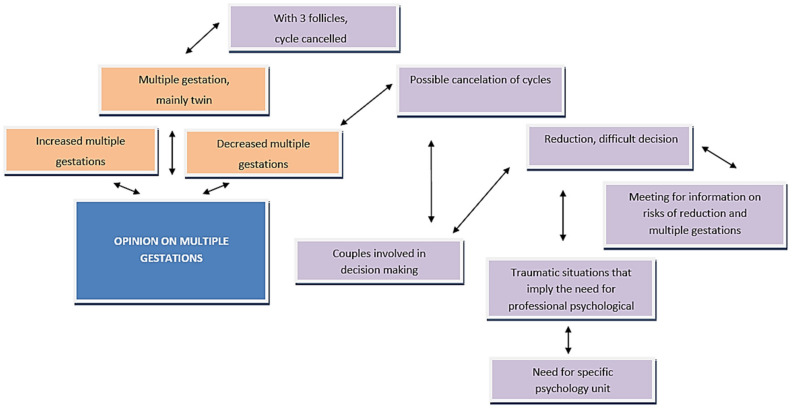
Opinion on the number of multiple gestations in recent years. Source: Own elaboration.

**Figure 2:**
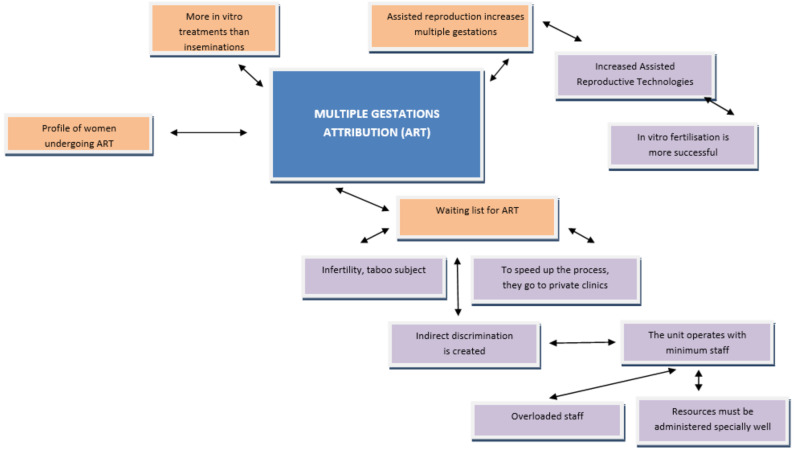
Multiple Gestation Attribution (ART). Source: Own elaboration; ART: Assisted Reproductive Technologies.

Figure 1 and Figure 2 show the different codes that make up the opinion section on the number of multiple gestations in recent years and the relationships between them.

In Figure 1, in the face of the possible situation of cycle reduction or multiple gestations, the importance of information meetings on reduction risks and multiple gestations is evident. Despite being much-desired pregnancies, both the mother and babies are exposed to risks that future parents should be aware of.

As for ART, reference is made to the need for a specific unit of psychology, as traumatic situations occur during the procedure that require psychological support. The main views of participants regarding the number of multiple gestations in recent years are illustrated in a cognitive map below.

**Figure 3:**
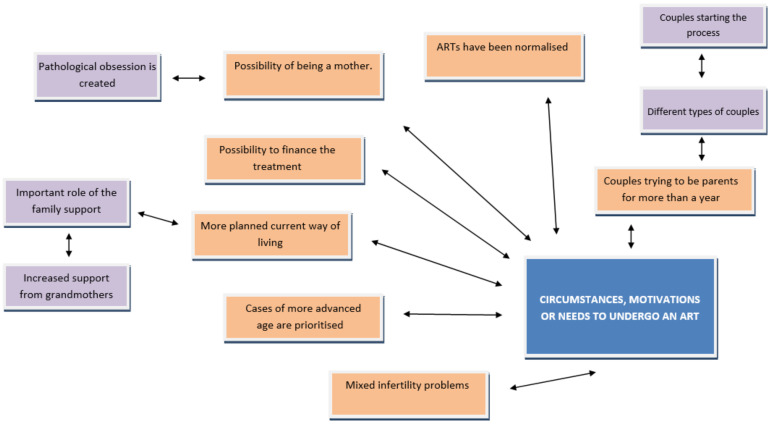
Circumstances, motivations or needs to undergo an ART. Source: Own elaboration; ART: Assisted Reproductive Technologies.

With regard to Figure 2, it must be highlighted that this includes a possible disadvantage, since, for assisted reproductive treatments, there is an important waiting list due to its high demand, thus slowing down the gestation process. With regard to the attribution of multiple gestations, the discourses of the participants can be summarised as follows:

Figure 3 shows a “cognitive map” based on the circumstances, motivations, and needs for assisted reproductive treatment. For this issue, the interviewees allude to a series of clear ideas to be discussed below:

The fundamental reason for couples to decide to undergo ART is because they have been trying to get pregnant for over a year with no success. However, there is also a circumstance in which there is no real infertility and the recommended waiting times have not been respected. The people interviewed allude to several reasons in this regard: assisted reproductive treatments have been standardised, they can be relatively easily financed, and above all, the constant programming of peoples’ lives, where motherhood is not left to chance and is highly planned according to women’s work wishes, in particular. This leads them to unfailingly delay pregnancy because there is no family reconciliation that actually safeguards their reproductive and career desires together.

### 3.2. Impact of Multiple Gestation on Women, Partners, Society, and the Health System

The collective of gynaecologists agrees on the serious emotional wear and tear of couples facing multiple gestations. Everyone, in the context of parenting, refers to the important psychic impact on mothers, because of the lack of time to internalise these emotional changes since all their energy and time are focused on this double upbringing. Stress is greatest in cases of prematurity, especially when there are differentiated needs between the two twins, and they feel they are providing unequal care.

There is a sense of critical emptiness and frustration in mothers once husbands regain their former life, while they remain totally immersed in parenting.

Fathers are often very involved in parenting and household chores, either because they have no choice when the mother is fully immersed in parenting or because they have had a hard time achieving that pregnancy and now they are enjoying a one-month parental leave that puts them at the forefront of the reality of parenting (although it is said that some fathers use these leaves for their personal time, often older men who are not involved).

There is agreement regarding the family support network being critical for coping positively.

The economic impact for couples is great, not only due to the investment made in reproductive treatments through private health, but also for what double upbringing implies. For the health system, there are economic demands as well, with the increasing maternal and perinatal morbidity associated with these gestations (Table 3).

**Figure 4:**
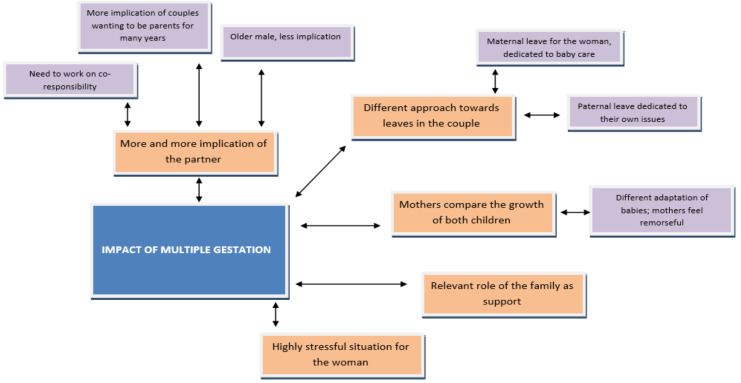
Impact of multiple gestation. Source: Own elaboration.

Through Figure 4, an analysis is made on the impact of multiple gestations at the various levels contemplated in the research according to the testimonies collected through these interviews.

This figure corroborates the above results, highlighting the relevance, as the study states, of the need for couples to have co-responsibility.

It also clearly shows the idea that older males are less involved than younger males, and eventually mentions the important role of family in supporting multiple gestation.

### 3.3. Follow-Up

All interviewees express the need for a specifically emotional, multidisciplinary and protocolised follow-up.

Although there is no differentiated protocol, midwives agree that their differentiated follow-up is much greater and that the primary-specialised inter-level relationship for proper gestation monitoring is satisfactory.

Infertility specialists refer to the fact that infertility is poorly sized, starting in primary care by family physicians, which delays and negatively impacts the follow-up of these couples, and emphasises the role of primary care midwives to be relevant and necessary for the successful monitoring of these gestations (Table 4).

### 3.4. Demands to the Health Administration

Midwives demand recognition of the differentiated care that they provide. Many post-night visits are not counted, so the problem of multiple gestations cannot really be sized and their impact on the system remains unnoticed. Public health gynaecologists understand that reproductive medicine is a second-rate specialty and that no investment is made in the resources needed for its proper development, which translates, for example, into unmanageable waiting lists for many women who require assisted reproductive treatment (Table 5).

**Figure 5:**
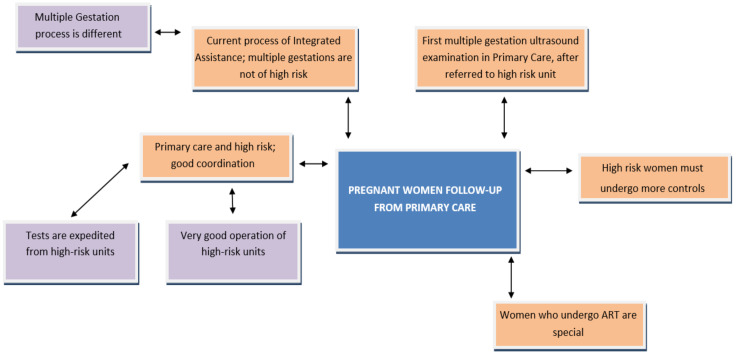
Follow-up to pregnant women from primary care. Source: Own elaboration; ART: Assisted Reproductive Technologies.

Figure 5 shows the perception that women with multiple gestations have of the follow-up made from primary care. It also reflects the differentiated care that midwives refer to in their follow-up, demanding to be recognised in order to make this work visible.

### 3.5. Suggestions

Among the suggestions provided by professionals to address the problem of multiple gestations are: raising awareness among women and their partners about the risks of multiple gestations for both them and their offspring; educating about the importance of the age of the first pregnancy and how that age will have a definite impact on women’s fertility; and generating multidisciplinary protocols that specifically deal with the emotional sphere and strengthen the economic support for these families (Table 6).

**Figure 6:**
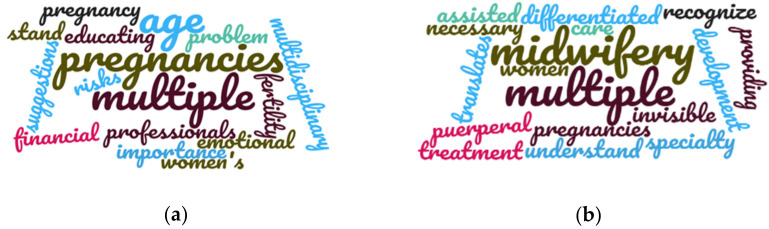
Tag cloud with critical points and improvement proposals. (**a**) Critical points. (**b**) Proposals for improvement.

The tag cloud (Figure 6) also shows critical points and improvement proposals after category analysis: health management demands and suggestions.

Following the analysis of the discourses and as a summary of the most important results, we emphasise that professionals stated that the current socioeconomic and legal context hinders a single embryonic transfer policy that decreases multiple gestation rates. They emphasised the importance of the psychic impact of such gestations on the couple, on the mother in particular, as well as the economic effect on families, health, and society in general. They expressed the need to create specific protocols to assist these gestations. The midwives, in particular, demanded for the health administration to recognise and support the differentiated care they perform with this type of gestation.

## 4. Discussion

Significant similarities have been found in the expressed opinions and professional demands, although they are a barely explored subject, since most offer a clinical vision of multiple gestation and, in particular, its relationship with ART [14].

Spain tops the ranking of European countries where the most cycles of assisted reproduction take place, and also ranks third globally below the United States and Japan [15].

The recommended multiple pregnancy rates after ART (in vitro fertilization) are below 10% [16]; therefore, over the past decade, the international scientific community has implemented transfer policies aimed at reducing multiple gestation rates by reducing the number of transferred embryos [17]. Currently, the recommendation is to carry out a single transfer in young patients of good prognosis as their pregnancy rate is not significantly affected, being, therefore, comparable with spontaneous pregnancies [18]. However, fertility societies currently raise the ethical dilemma between the right of patient autonomy and the recommendations of performing single transfer to avoid the risks associated with multiple gestation, which is a strong moral, socioeconomic, and scientific debate still unresolved. These data coincide with our research, where the opinion of professionals in this regard was: “Legal protection along with the socio-economic circumstances of couples make elective transfer difficult even if recommended”.

In Spain, since 2006 and under Law 14/2006 [19], the transfer/cycles of embryos were limited to a maximum of three. This law remains in force today and, under its protection, the percentage of double transfers continues to account for more than half of the total transfers made in the country. This is reflected in the statistical report of 2017 of the Spanish Society of Fertility on assisted reproductive technologies [20]; 60.3% of the transfers were of two embryos that, in 23.5%, resulted in multiple gestation, although since 2015, the embryo transfer policy is to increase the number of unique transfers. This is largely explained by the pressure that health professionals receive on behalf of patients requesting double transfers as they are considered more profitable in terms of their socioeconomic and work context [21,22].

The Guide to Assisted Human Reproduction in the Public Health System of Andalusia [23] contains a specific section on the human and material resources necessary for the operation of these centres. However, this section does not cite the psychological profile as part of the equipment necessary to serve the applicants for these services. However, on the other hand, the testimonies contained in our results show the importance of this group for the correct care of users of ART, demanding its presence in public health as it is in private assistance.

Treatments of ART in public health are free of charge, resulting in such high demand that waiting lists are sometimes impossible to cope with by the applicants. This causes a leak to private assistance where such waiting lists do not exist, even if treatments come at a very high cost. This is a common complaint that is reflected in the testimonies of participating gynaecology professionals who demand more economic investment for public centres.

Currently, in Spain, the mean age for motherhood is 32.25 years and the mean number of children per woman is 1.31 [24]. In the lives of women, motherhood becomes a complement to their profession, which is now a life project, causing changes in motherhood patterns with a delayed and reduced number of sons or daughters [25]. Late motherhood beyond the age of 35 is the most decisive factor in infertility, where multiple gestation after ART is more than probable. More than 50% of patients come from fertility treatments and older maternal age is more common [26]. In this same line, the literature highlights the important implications of the age of 35 for fertility and for the added risks of multiple gestation [27]. This idea is reflected in our results as the people interviewed express the delay of motherhood as one of the main causes for the use of ART, a delay related, among other causes, to the professional aspirations of women.

Multiple gestations account for 10% of overall perinatal mortality and overall twin mortality is 5–10 times higher than in simple gestations, with higher prematurity rates, resulting in a large impact on the morbidity of descendants. Prematurity is reflected in our research as a source of added stress in the context of twin pregnancy. In addition, the overall maternal mortality associated with multiple births is 2.5 times higher than that of single births [28]. In parallel with the drastic increase in multiple gestations in recent decades, there has also been an increase in caesarean rates, largely due to the belief that these improve perinatal results. In fact, elective caesarean is the most common form of birth in twin gestations [29,30].

The importance of support networks and the psychic impact of multiple gestations is highlighted in the testimonies of the interviewees and is, thus, reflected in the literature, where we find that, regardless of the family situation or structure, support networks are fundamental to the maternity process and other daily tasks [31]. However, in the case of multiple gestations, there is almost twice the risk of postpartum depression and depressive symptoms, compared to single gestations [32]. Likewise, the psychological impact on the relationship is significant and the mental health of these parents is worse [33].

This impact on the emotional sphere is exacerbated by the economic difficulties that already arise at the start of such gestations in order to resort to ART, and then, upbringing. For families, socioeconomic costs are approximately 4 to 11 times higher in twin gestations than in simple gestations [34]. Therefore, economic burdens, as well as the potential to reduce the quality of life of twins, need careful evaluation.

In addition, due to the increased risk of complications, women with multiple pregnancies need more health monitoring and greater contact with health care and their professionals, which will have an impact on health system resources [35]. On this line, the people interviewed allude to the economic impact of multiple gestations on the family, but also on the health system and society in general.

The Anglo-Saxon context reflects the need for differentiated follow-up for multiple gestations [36], which is an opportunity to ensure that guidelines reflect best practices and women receive the best possible care. In Andalusia, the follow-up of all gestations in the Autonomous Community is carried out through a care programme which is defined and delimited by the Integrated Care Process (PAI, for its acronym in Spanish) of Pregnancy, Childbirth, and Postpartum (EPP, for its acronym in Spanish). However, this process does not consider the special needs for emotional support that arise in cases of multiple gestations.

Additionally, although there are concrete guidelines for the management of such gestations by some scientific societies such as the Spanish Society of Obstetrics and Gynaecology (SEGO) [37], these do not contain general recommendations adapted to this type of gestation or the need for greater emotional support. In contrast, our results do illustrate the need for such circumstances when the great psychic impact of twin gestations manifests in most testimonies. However, these particular needs, in the case of midwives, who are regarded as developing a key role in the preparation of mothers and fathers of twins [38], are being met even though they do not have any official protocol in this regard. Such would be the case of advancing vaginal BGS screening. This is reflected in the “Protocol of Assistance to Pregnancy and Childbirth of Multiple Gestations” of the Clinic Hospital in Barcelona, though this is not a scientific society, but a specific hospital [39].

In the region of Andalusia (Spain), the electronic medical history support system (Diraya) integrates all the health information of the people assisted in the health centres, and also serves as the management of the health system. In the case of the postpartum visits, only those made the first ten days after delivery are recorded, even though in the case of multiple gestations, midwives continue to assist and offer care beyond this date. In contrast, the current literature reflects the enormous importance that registered midwives can have on maternal satisfaction, as well as the need to support their professional role and positively impact teamwork, organisational processes, and research [40,41]. The testimonies of the interviewed midwives point out the current configuration of this programme as a great disadvantage, because it does not allow the differentiated attention they are giving to these pregnant women to be faithfully viewed.

Although Organic Law 3/2007 [42] for the effective equality of men and women, in article 44, determines the rights to reconcile family and work life, even if they have some legal protection, many women encounter serious problems related to work leaves during fertility treatments or in the case of pregnancy. In short, as Gracia-Maroto et al. already stated, the increase in fertility rates implies the transformation of different social aspects related to restructuring productive and reproductive work, the development of social services, enhancement of institutional aid, and the revision and improvement of health care policies [43].

## 5. Limitations

There are a number of limitations in the present study. First, it is worth considering the exploratory nature of this research and the participation of only eight subjects. This is why our results have limited generalisation. For future research, collecting other significant opinions, such as those of couples undergoing ART and mothers with multiple gestations, is being considered.

On the other hand, in the current literature, no article has been found that has studied the testimony of the participants selected in the research at hand on multiple gestations and their relationship with ART. This limitation is understood as an opportunity to identify new gaps in the literature and, consequently, new research that allows a greater body of knowledge on the subject of research and improved practice.

Another limitation is the time available to investigate the issue; since the interviews were conducted in the workplaces of the participants, we adjusted to agenda gaps and moments of rest which, in some cases, did not allow the interviews to expand as much as desired.

The study only reflects the discourse of the collectives of gynaecology and midwifery, while this impact could have included other health actors such as primary care physicians, who are directly referred to by gynaecologists, thus providing complementary results.

## 6. Conclusions

For the collective of gynaecology, it is important to apply single embryo elective transfer policies in cases of ART. This would allow the rates of multiple gestations after the use of these techniques to be maintained at the recommended percentages and control the clinical and economic impact of multiple gestations.

Midwives stress the need to improve clinical documentation registration systems to be a true reflection of the follow-up needs posed by multiple gestations.

For both professional groups, the socioeconomic implications and impact on maternal–foetal and neonatal health must be considered by political/health authorities and, also in this regard, educate future parents.

As for the practical implications of this study, progress should be made in protocols that contribute to better care and monitoring of women’s emotional health in particular, and couples in general, optimising clinical decisions and promoting safe, quality, and lower cost care. In addition, developing sociohealth policies that lead to co-responsible reconciliation measures that favour women having one pregnancy at a time is necessary (although, moral, socioeconomic, and scientific debates are still unresolved).

More research is needed to investigate the opinions and experiences of other health professionals and couples to delve into the factors that prevent the complex problem of multiple pregnancy from actually being determined today.

## Figures and Tables

**Table 1:** Profiles of interviewees.

Professionals	Time of Service	Sex	Occupation	Age (years)	Place of Work
P 1	15 years	Female	Midwife	58	PC
P 2	30 years	Male	Midwife	55	PC
P 3	20 years	Female	Midwife	62	PC
P 4	30 years	Female	Midwife	57	PC
P 5	12 years	Male	Gynaecologist	62	Public ART Unit
P 6	7 years	Female	Gynaecologist	47	Public ART Unit
P 7	3 years	Female	Gynaecologist	32	Private ART Unit
P 8	24 years	Male	Gynaecologist	53	Private ART Unit

ART, assisted reproductive technologies; PC, primary care.

**Table 2:** Current trend of multiple gestations (embryo transfer policies).

Current Trend for Multiple Gestations (Embryo Transfer Policies)
*“Of course. The big increase has come since assisted reproductive…” (p2).* *“Transferring three embryos is a bad practice, by all standards, even twin pregnancies are in question. But of course, as by law, it’s not illegal… That is, there are people who do it and are protected by law, and even there are some… a unique embryo transfer policy is being made in many…” (p6).* *“It’s an individual ethical-personal issue, so I think the first thing is primary prevention, not transferring more than two embryos. In fact, the increasing trend is to transfer one” (p7).* *“The law in Spain… only three was authorised and this did a lot to correct multiple pregnancies and, currently, the goal is to reduce even the gestation of two” (p8).* *“Here is a very clear economic approach, everything achieved by that perverse incentive, which is that if I have two children at once, all the better, I have my family and once for in a year or two, I have already solved the problem and I return to my work and personal life” (p8).* *“But you understand that, for many women that age, they cannot stop working three times to have three children, with the maternal leaves this implies and everything it entails. Unfortunately, there is still a lot of machismo and micro-machismo, so many women in their jobs will not tolerate two pregnancies or three” (p6).* *“I also believe that if motherhood were better cared for with regard to maternity leave… But if there is no reconciliation, you reach 42 years old and you want to have everything arranged, for the nursery and all these things, you have no choice but to leave at 40–42 years. But if you have more facilities such as governments to help you, more reconciliation so that the father can access a good paternal leave… because perhaps you raise it younger because, well, the government helps me in this period, and my husband is also there” (p7).*

**Table 3:** Impact of multiple gestations on women, couples, society, and the health system.

Impact of Multiple Gestations on Women, Couples, Society, and the Health System
*“To the woman, very emotionally, the whole issue of morbidity, postpartum depressions, any kind of psychiatric morbidity, everything is going to shoot up” (p1).* *“All kinds of couple crises can be seen in matters of reproduction and infertility, because it is a kind of vital crisis that can sometimes be very disturbing” (p8).* *“It’s not only physiological stress, it is also a psychic and economic stress. So, this strains the couple” (p8).* *“When they are premature… you have to spend 3 or 4 weeks at hospital… and that marks women a lot, huh, that… they end up shattered, because they are coming and going all the time…” (p2).* *“They get exhausted and see that all the emotional resources are allocated to the children, they are the only ones who have the focus on them and they somehow recover “a life of man” (p8).* *“Of course, prematurity has this problem of these children, their early neonatal but also long-term intellectual performance” (p8).* *“In fact, there is a psychologist at the clinic who recently emailed us that all women undergoing treatment should do therapy” (p7).* *“But, come on, males put up with it worse. And it is true that we live in a very sexist society, very sexist, it is still very sexist and… and women… I increasingly admire women for their ability to hold on” (p6).*

**Table 4:** Follow-up of multiple gestations.

Follow-Up of Multiple Gestations
*“You have to further control: these women… you have to follow up much more. So, it’s pregnancies that you have to control them more…” (p1).* *“When I see that it’s a twin pregnancy, I like to do the home visit, see the conditions these kids are going to be in” (p2).* *“Protocols tell the same old thing, but then you have to have your internal protocol that we all have that is given by experience” (p3).* *“I do. Normally, you’re required a post-night visit by the administration. If I, to a normal woman with one child, I don’t have enough because I give her an appointment her and then I do it again to remove the stitches, then I give these women five appointments because with the tits they need more care” (p4).* *“And me, for instance, in twin pregnancies, streptococcus [¿?] I perform it a bit earlier, on my own account. This is not protocolised, but I know this woman needs it (p2)”.* *“Then I start preparing these women separately, a little earlier; in the remaining pregnant women, I start at week 27; in those, I start at week 24 or 23, so… just in case” (p2).* *“They need the midwife more than the… that norm… patients with a single pregnancy” (p6).*

**Table 5:** Demands to the health administration.

Demands to the Health Administration
*“More midwives must be demanded considering the women” (p3).* *“I think with the twins there should be more visits because, besides, many are C-sections, and coming with the two children is more complicated. What’s more, the visits I make are for twins or if there has been some complication in the postpartum” (p4).* *“To contemplate that, if it is a twin pregnancy, the ten days should not count because since the children are admitted, that woman does not come until twenty days later, and now you see her at 20 days and the visit does not count. So, this is very hard, but in public health it’s a disaster” (p4).* *“Yes, yes, they consider it to be… a slightly absurd specialty,… should be more recognised and that goes through the economic issue, above all” (p6).* *“And in the end, they choose to go to a private clinic to speed things up, because in the public system, waiting times are often unsustainable, then more investment to speed up times” (p7).* *“Like public medicine, I believe that… that does not live up to the circumstances, most of the… couples have to resort to the private ones that take less time, and couples are taken care of more psychologically” (p5).*

**Table 6:** Suggestions.

Suggestions
*“To raise patients’ awareness of the risks of a multiple pregnancy for both her and the foetuses. And I think that’s everybody’s job.” (p7).* *“Also, as primary prevention is to raise awareness of the age of the first pregnancy. To know that from the age of 35 fertility is reduced exponentially and that from the age of 38, it is much more reduced” (p7).* *“Then, we should find a positive incentive for these women to take on gestations one at a time” (p8).* *“No. Not at all. No. Someone who, at any given time, the moment comes… and have free time and say “Well, I’m going to get ready…”, and do a programme and mess with the psychologists, and mess with the midwives, and with the gynaecologists, and does a programme, and make a booklet and a brochure and a downloadable PDF and… whatever you want…” (p1).* *“The thing would be to have a specific unit of… of… psychology of… to support these couples, that would be clear, that would be great” (p5).* *“The social and economic aspect is where we should find an economic support network for couples, for the financing of procedures and cycles, which would reduce the stress of having multiple pregnancies, and this would be the most efficient way to reduce the number of embryos and multiple gestations” (p8).*

## Data Availability

All data are available within this article and its supplementary file.

## Supplementary Materials

The following are available online at https://www.mdpi.com/article/10.3390/ijerph18116031/s1. Supplementary Material S1, Planned Script for the Interview: Midwives; Supplementary Material S2, Planned Script for the Interview: Assisted Reproduction Unit Professionals.

## References

[1] González-Mesa E., Cazorla O., González-Valenzuela M.J. (2015). The epidemic of twins: The challenge in obstetrics and gynecology. EMJ Reprod. Health.

[2] Heino A., Gissler M., Hindori-Mohangoo A.D., Blondel B., Klungsøyr K., Verdenik I., Mierzejewska E., Velebil P., Sól Ólafsdóttir H., Macfarlane A. (2016). Variations in Multiple Birth Rates and Impact on Perinatal Outcomes in Europe. PLoS ONE.

[3] Di Paola R., Garzon S., Giuliani S., Laganà A.S., Noventa M., Parissone F., Zorzi C., Raffaelli R., Ghezzi F., Franchi M. (2018). Are we choosing the correct FSH starting dose during controlled ovarian stimulation for intrauterine insemination cycles? Potential application of a nomogram based on woman’s age and markers of ovarian reserve. Arch. Gynecol. Obstet..

[4] Papler T.B., Bokal E.V., Zmrzljak U.P., Stimpfel M., Laganà A.S., Ghezzi F., Jančar N. (2019). PGR and PTX3 gene expression in cumulus cells from obese and normal weighting women after administration of long-acting recombinant follicle-stimulating hormone for controlled ovarian stimulation. Arch. Gynecol. Obstet..

[5] Alemany-Lasheras F., Amezcua-Martínez M., Aparcero-Bernet L., Arroyo-Rodríguez A., Calvo-Cabrera I., Lancharro-Tavero I. (2015). Memoria de la asignatura Trabajo Fin de Grado. Centro Universitario de Enfermería “San Juan de Dios”. Universidad de Sevilla. Bibl. Las Casas.

[6] Goncé A., Boguña J., Marimon E., Muñoz M., Palacio M., Martínez J. (2015). Protocolo Asistencia al Embarazo y Parto de Gestaciones Múltiples.

[7] Committee on Practice Bulletins—Obstetrics, Society for Maternal–Fetal Medicine (2016). Practice Bulletin No. 169: Multifetal Gestations: Twin, Triplet, and Higher-Order Multifetal Pregnancies. Obs. Gynecol..

[8] Qazi G. (2011). Obstetric and perinatal outcome of multiple pregnancy. J. Coll Physicians Surg. Pak..

[9] Qin J., Wang H., Sheng X., Xie Q., Gao S. (2016). Assisted reproductive technology and risk of adverse obstetric outcomes in dichorionic twin pregnancies: A systematic review and meta-analysis. Fertil. Steril..

[10] Bladilo A., Torre N., Herrera M. (2017). Las técnicas de reproducción humana asistida desde los derechos humanos como perspectiva obligada de análisis. IUS.

[11] Benute G., Nozzela D., Prohaska C., Liao A., De-Lucia M., Zugaib M. (2013). Twin pregnancies: Evaluation of major depression, stress and social support. Twin Res. Hum. Genet..

[12] Chambers G.M., Lee E., Hansen M., Sullivan E.A., Bower C., Chapman M. (2014). Hospital costs of multiple-birth and singleton-birth children during the first 5 years of life and the role of assisted reproductive technology. JAMA Pediatr..

[13] Palacios-Ceña D., Corral-Liria I. (2010). The basics and development of a phenomenological research protocol in nursing. EnfermeríaIntensiva.

[14] Chien P. (2020). Multiple pregnancy and assisted conception treatment. BJOG An Int. J. Obstet. Gynaecol..

[15] Rivas A., Álvarez C., Jociles M. (2018). La intervención de ‘terceros’ en la producción de parentesco: Perspectiva de los/as donantes, las familias y la descendencia. Un estado de la cuestión. Rev. Antropol. Soc..

[16] Jacklin P., Marceniuk G. (2018). A Report by the National Guideline Alliance about Twin Pregnancy Costing Commissioned by: The Human Fertilisation and Embryology Authority, the British Fertility Society, the Multiple Births Foundation and Fertility Network UK. https://www.hfea.gov.uk/media/2650/nga-twin-pregnancy-costing-final.pdf.

[17] McLernon D.J., Harrild K., Bergh C., Davies M.J., De Neubourg D., Dumoulin J.C.M., Gerris J., Kremer J.A., Martikainen H., Mol B.W. (2011). Clinical effectiveness of elective single versus double embryo transfer: Meta-analysis of individual patient data from randomised trials. BMJ.

[18] Tobias T., Sharara F., Franasiak J., Heiser P., Pinckney-Clark E. (2016). Promoting the use of elective single embryo transfer in clinical practice. Fertil. Res. Pract..

[19] (2006). Ley 14/2006 de 26 de mayo sobre técnicas de reproducción asistida. Boletín Oficial del Estado (BOE).

[20] Comisión Nacional de Reproducción Humana Asistida (2017). Informe Estadístico de Técnicas de ReproduccionAsisitda.

[21] Ezugwu E., der-Burg S. (2015). Debating elective single embryo transfer after in vitro fertilization: A plea for a context sensitive approach. Ann. Med. Health Sci. Res..

[22] Adashi E., Gleicher N. (2017). Is a blanket elective single embryo transfer policy defensible?. Rambam Maimonides Med. J..

[23] Junta de Andalucía (2020). Reproducción Humana Asistida en el Sistema Sanitario Público de Andalucía.

[24] Instituto Nacional de Estadística (2019). INE. Indicadores de Fecundidad. Registrosnacionales..

[25] Román E., Coca A., García E. (2017). Maternidad y conciliación laboral: ¿mito o realidad?. Enfermería Docente.

[26] Kawwass J., Badell M. (2018). Maternal and fetal risk associated with assisted reproductive technology. Obstet. Gynecol..

[27] Toneut C.M., García M.E.G., Vega A.M., Fernández R.B., Arechavaleta N.M., Arechavaleta A.M. (2017). Maternal and perinatal outcomes in pregnant women with advanced maternal age. Rev. Cuba. Obstet. y Ginecol..

[28] Santana D.S., Silveira C., Costa M.L., Souza R.T., Surita F.G., Souza J.P., Mazhar S.B., Jayaratne K., Qureshi Z., Sousa M.H. (2018). Perinatal outcomes in twin pregnancies complicated by maternal morbidity: Evidence from the WHO Multicountry Survey on Maternal and Newborn Health. BMC Pregnancy Childbirth..

[29] Reitter A., Daviss B.A., Krimphove M.J., Johnson K.C., Schlößer R., Louwen F., Bisits A. (2018). Mode of birth in twins: Data and reflections. J. Obstet. Gynaecol..

[30] Ellwood D. (2020). Caesarean secion births for twins: Rational choice, or a non-evidence-based intervention that may cause harm?. Med. J. Aust..

[31] Díez M., Morgado B., González M. (2017). El apoyo social y la satisfacción vital, factores clave en el caso de las madres adptivas solas. Apuntes Psicol..

[32] Van den Akker O., Postavaru G., Purewal S. (2016). Maternal psychosocial consequences of twins and multiple births following assisted and natural conception: A meta-analysis. Reprod. Biomed. Online.

[33] Wenze S., Battle C., Tezanos K. (2015). Raising multiples: Mental health of mothers and fathers in early parenthood. Arch. Women’s Ment Health.

[34] Lemos E., Zhang D., Van Voorhis B., Hu X. (2013). Healthcare expenses associated with multiple vs singleton pregnancies in the United States. Am. J. Obstet. Gynecol..

[35] National Collaborating Centre for Women’s and Children’s Health (2011). Commissioned by the National Institute for Health and Clinical Excellence. Multiple Pregnancy: The Management of Twin and Triplet Pregnancies in the Antenatal Period. https://www.nice.org.uk/guidance/ng137/evidence/september-2011-full-guideline-pdf-6901736510.

[36] National Institute for Health and Care Excellence (2019). NICE Guideline. Twin and Triplet Pregnancy. www.nice.org.uk/guidance/ng137.

[37] (2016). SEGO (Sociedad Española de Ginecología y Obstetricia). Guía de asistencia práctica. Dichorionic twin pregnancy. Prog. Obstet. Ginecol..

[38] Carrick-Sen D., Steen N., Robson S. (2014). Twin parenthood: The midwife’s role—A randomised controlled trial. BJOG.

[39] Ponce J., Bennasar M., Muñoz M., Palacio M., Crovetto F., Boguña J. (2021). Protocolo Asistencia al Embarazo y Parto de Gestaciones Múltiples.

[40] Griffith R. (2016). Records: What to include. Br. J. Midwifery.

[41] Kerkin B., Lennox S., Patterson J. (2018). Making midwifery work visible: The multiple purposes of documentation. Women Birth.

[42] De España G. (2007). Ley Orgánica 3/2007 de 22 de marzo para la igualdad efectiva de hombres y mujeres. Boletín Oficial del Estado (BOE).

[43] Maroto-Navarro G., Castaño-López E., García-Calvente M., Hidalgo-Ruzzante N., Mateo-Rodríguez I. (2009). Paternity and HealthServices. Qualitative Research on Men’s experiences during Pregnancy, Delivery and Postpartum of theirs Partners. Rev. Española de SaludPública.

