# Supplementary material for: Multiple Gestations and Assisted Reproductive Technologies: Qualitative Study of the Discourse of Health Professionals in Spain
Source: Int J Environ Res Public Health. 2021 Jun 3;18(11):6031. doi: 10.3390/ijerph18116031 (PMC8200015; doi:10.3390/ijerph18116031)
Supplement: Supplementary file 1 [file ijerph-18-06031-s001.zip › ijerph-1218026-supplementary.pdf]

International Journal of
*Environmental Research and Public Health*

**Supplementary Material S1.** Planned Script For The Interview: Midwives

**1. Multiple Current Trends.**

1.1 What is your opinion on the number of multiple gestations in recent years?

1.2 What do you attribute it to? Assisted Reproductive Technologies? (origin).

1.3 What do you think are the circumstances, motivations, or needs to undergo Assisted Reproductive Technologies? (may include haste and publicity).

1.4 In your opinion, what are the motivations for delaying motherhood?

**2. Impact.**

2.1 What is the impact of multiple gestation on the couple, family, and/or society?

2.2 What is the impact of multiple gestation on women's physical and emotional health?

**3. Follow-Up.**

3.1 What strengths and weaknesses do you think follow-up to pregnant women from primary care present?

3.2 How do you value emotional follow-up for women and couples undergoing Assisted Reproductive Technologies?

3.3 If valued negatively not, what do you think this is due to: lack of resources, little involvement of professionals...?

3.4 And, regarding this follow-up in the postpartum, in your experience, what would you highlight and why?

3.5 What is your opinion regarding stress during this period?

**4. Demands to the Health Administration.**

4.1 How is the administration's management of multiple gestation women?

4.2 What role are we, professionals, taking on in this regard?

4.3 Do you have any requests in this regard?

**5. Suggestions.**

5.1 Would you like to add anything else on the subject?

5.2 Is there anything I have not asked you? What do you think it is?

**Supplementary Material S2.** Planned Script for the Interview: Assisted Reproduction Unit Professionals.

**1. Multiple Current Trends.**

1.1 What is your opinion on the number of multiple gestations in recent years?

1.2 What do you attribute it to? Assisted Reproductive Technologies? (origin).

1.3 How do you address the issue of selective reduction?

1.4 What do you think are the circumstances, motivations, or needs to undergo Assisted Reproduction Therapy? (may include haste and publicity).

1.5 In your opinion, what are the motivations for delaying motherhood?

**2. Impact.**

2.1 What is the impact of treatments on the couple, at the work level and/or at the social level?

2.2 What is the impact of multiple gestation on the couple, family, society, and women themselves?

**3. Follow-Up.**

3.1 How do you value emotional follow-up for women and couples undergoing Assisted Reproduction Therapy?

3.2 If not, what do you think this is due to: lack of resources, little involvement of professionals...?

3.3 And, regarding this follow-up in the postpartum, in your experience, what would you highlight and why?

3.4 What is your opinion regarding stress during this period?

**4. Demands to the Health Administration.**

4.1 How is the administration's management of multiple gestation women?

4.2 What role are we, professionals, taking on in this regard?

4.3 Do you have any requests in this regard?

**5. Suggestions.**

5.1 Would you like to add anything else on the subject?

5.2 Is there anything I have not asked you? What do you think it is?
